# Propensity score-matched analysis of systemic chemotherapy versus salvage hysterectomy for persistent cervical cancer after definitive radiotherapy/concurrent chemoradiotherapy

**DOI:** 10.1186/s12885-020-07672-w

**Published:** 2020-11-30

**Authors:** Munetaka Takekuma, Fumiaki Takahashi, Seiji Mabuchi, Wataru Kudaka, Koji Horie, Mariko Ikeda, Ayumi Shikama, Akira Mitsuhashi, Shoji Nagao, Shiro Suzuki, Mika Mizuno, Shin Nishio, Hideki Tokunaga, Yukinobu Ota, Takahiro Kasamatsu, Ryo Kitagawa, Takafumi Toita, Hiroaki Kobayashi, Mitsuya Ishikawa, Nobuo Yaegashi

**Affiliations:** 1 Department of Gynecology, Shizuoka Cancer Center, 1007 Shimonagakubo, Nagaizumicho, Suntogun, Shizuoka, 411-8777 Japan; 2 Department of Information Science, Iwate Medical University, Idaidori, Yahabacho, Shiwagun, Iwate, 028-3694 Japan; 3 Department of Obstetrics and Gynecology, Osaka University Graduate School of Medicine, 202 Yamadaoka, Suita, Osaka, 565-0871 Japan; 4 Department of Obstetrics and Gynecology, Graduate School of Medical Science, University of the Ryukyus, 207 Uehara, Nishiharacho, Nakagamigun, Okinawa, 903-0125 Japan; 5 Department of Gynecology, Saitama Cancer Center, 818 Komuro, Inamachi, Kitaadachigun, Saitama, 362-0806 Japan; 6 Department of Gynecology, Kanagawa Cancer Center, Nakaonaga, Asahiku, Yokohama, Kanagawa 241-0815 Japan; 7 Department of Obstetrics and Gynecology, Faculty of Medicine, University of Tsukuba, Tennodai, Tsukuba, Ibaraki, 305-8575 Japan; 8 Departments of Reproductive Medicine, Graduate School of Medicine, Chiba University, 1-8-1 Inohana, Chuoku, Chiba, Chiba 260-8677 Japan; 9 Department of Gynecologic Cancer, Hyogo Cancer Center, 13-70 Kitaozicho, Akashi, Hyogo 673-8558 Japan; 10 Department of Obstetrics and Gynecology, Nagoya University Graduate School of Medicine, 65 Tsurumaicho, Showaku, Nagoya, Aichi 466-8550 Japan; 11 Department of Gynecology, Aichi Cancer Center Hospital, Kanokoden, Chikusaku, Nagoya, Aichi 464-8681 Japan; 12 Department of Obstetrics and Gynecology, Kurume University School of Medicine, 67 Asahicho, Kurume, Fukuoka, 830-0011 Japan; 13 Department of Obstetrics and Gynecology, Tohoku University Hospital, Seiryo-machi, Aobaku, Sendai, Miyagi 980-8574 Japan; 14 Department of Gynecology, Osaka International Cancer Institute, 3-1-69, Otemae, Chuoku, Osaka, Osaka 541-8567 Japan; 15 Department of Obstetrics and Gynecology, Tokyo Metropolitan Bokutoh Hospital, 4-23-15 Kotobashi, Sumidaku, Tokyo, 130-8575 Japan; 16 Department of Gynecology and Obstetrics, Tohoku Medical and Pharmaceutical University, 4-4-1 Komatsushima, Aobaku, Sendai, Miyagi 981-8558 Japan; 17 Radiation Therapy Center, Okinawa Chubu Hospital, 281 Aza-miyazato, Uruma, Okinawa, 904-2293 Japan; 18 Department of Obstetrics and Gynecology, Faculty of Medicine, Kagoshima University, 8-35-1 Sakuragaoka, Kagoshima, Kagoshima 890-8544 Japan; 19 Department of Gynecology, National Cancer Center Hospital, 5-1-1 Tsukiji, Chuoku, Tokyo, 104-0045 Japan

**Keywords:** Uterine cervical Cancer, Persistent diseases, Salvage hysterectomy, Radiotherapy, Survival

## Abstract

**Background:**

The aim of the current study was to evaluate oncologic outcomes of patients who were treated with salvage hysterectomy (HT), compared to systemic chemotherapy (CT) for persistent cervical cancer after definitive radiotherapy (RT)/ concurrent chemoradiotherapy (CCRT).

**Methods:**

Patients with persistent cervical cancer treated with definitive RT/CCRT at 35 institutions from 2005 to 2014 were reviewed retrospectively (*n* = 317). Those who underwent a HT for persistent cervical cancer after definitive RT/CCRT were matched with propensity scores for patients who underwent systemic CT. Oncologic outcomes between the two groups using a propensity score matched–cohort analysis were compared.

**Results:**

A total of 142 patients with persistent cervical cancer after definitive RT/CCRT were included after matching (HT: 71, systemic CT: 71). All background factors between HT and CT groups were well balanced. Median overall survival was 3.8 and 1.5 years in the HT and CT groups, respectively (*p* = 0.00193, hazards ratio [HR] 0.41, 95% confidence interval [CI] 0.23–0.73), Increasing residual tumor size was significantly associated with a high incomplete resection rate (*p* = 0.016, Odds Ratio 1.11, 95%CI 1.02–1.22). Severe late adverse events occurred in 7 patients (9.9%) in the HT cohort.

**Conclusion:**

The current study demonstrated that, when compared to systemic CT, the adoption of salvage HT for patients with persistent cervical cancer after definitive RT/CCRT reduced mortality rate by about 60%. This indicates that salvage HT could be curative treatment for those patients. Further prospective clinical trials with regard to salvage HT after RT/CCRT are warranted.

**Supplementary Information:**

The online version contains supplementary material available at 10.1186/s12885-020-07672-w.

## Background

The survival of patients with cervical cancer has improved over recent decades due to improvements in screening, which allows for the detection and removal of precancerous lesions, generalization of vaccination against human papilloma virus (HPV), and the improved efficacy of anticancer therapeutics. However, there were approximately 12,820 new cases and 4210 cervical cancer-associated deaths in the US in 2017 [1].

Early stage cervical cancer can be cured with surgery, while concurrent chemoradiotherapy (CCRT) is accepted as the standard treatment for patients with locally advanced cervical cancer [2]. However, the 5-year-overall survival (OS) of locally advanced cervical cancer remains approximately 70% [3]. One fifth of patients with stage III-IVA were reported to have persistent disease after definitive CCRT [4], and these patients have a poor prognosis because cisplatin-based chemotherapy (CT), which is the treatment of choice, is not be effective for those patients [5]. The development of novel treatments for these patients is therefore needed, as it would address an un-met medical need.

Salvage hysterectomy (HT) is considered curative treatment; however, very little data exists showing the survival advantage of HT over standard systemic CT. Many guidelines do not have definitive recommendations regarding the treatment of residual tumors of the uterine cervix after radiotherapy (RT)/CCRT, although pelvic exenteration or radical HT is raised as an optional treatment for patients with local recurrence of central disease [6–8].

Ota, et al. showed that HT significantly improved disease-specific 5-year survival rates for patients with residual tumor after RT (rates were 68.6 and 14.5%, with and without HT respectively, *p* = 0.0001) [9]. However, safety is a critical issue for those patients with persistent or recurrent disease after RT or CCRT who may be eligible for salvage surgery. This is because severe complications, including postsurgical death, have been reported with relatively high frequency (16–25% of adverse events ≥ grade 2) [9–15].

The aim of the current study is to evaluate oncologic outcomes of patients with persistent cervical cancer after definitive RT/CCRT who were treated with salvage HT, compared to systemic CT using a propensity score analysis.

## Methods

### Setting and subjects

We conducted a retrospective cohort study using data from 35 academic and/or regional cancer centers that belong to the Japan Clinical Oncology Group (JCOG), the largest Japanese cooperative group funded primarily by the National Cancer Center Research and Development Fund. The protocol was approved by the institutional review board of each participating institution, and the need for written informed consent from each patient was waived.

Patients were identified by using an institutional database at each participating institution. All patients were treated with definitive RT/CCRT between January 2005 and December 2014. To be included in this study, patients had to meet all of the following conditions: (1) persistent cervical cancer clinically diagnosed after definitive RT/CCRT; (2) salvage treatment with HT, excluding pelvic exenteration (PE), or systemic CT within a year after completion of definitive RT/CCRT; (3) clinical stage IB1, IB2, IIA1, IIA2, IIIA, IIIB and IVA at initial treatment, according to the staging system of the International Federation of Gynecology and Obstetrics (FIGO); (4) less than 75 years of age at the diagnosis of persistent cervical cancer; (5) histological confirmation of the primary site; (6) histological type of squamous cell carcinoma (SCC), adenocarcinoma (AC), or adenosquamous cell carcinoma (ASC).

### Data elements

For eligible patients, we noted data regarding the following clinical and pathological details: patient demographics, treatment methods of definitive RT/CCRT, status of persistent cervical cancer, treatment contents and adverse events of salvage HT or systemic CT, and survival outcomes. For patient demographics, patient age and performance status (PS) at diagnosis of persistent cervical cancer, histological type, FIGO stage, tumor diameter, presence or absence of invasion to corpus lesion and number of lymph node metastasis at initial treatment were collected. Treatment methods of definitive RT/CCRT were evaluated based on total RT dose to the cervical lesion, presence or absence of intracavitary brachytherapy (ICBT) and concurrent chemotherapy. All treatments were normalized to biologically equivalent total doses in 2 Gy per fraction (EQD2) using the linear-quadratic model with α/β = 10 Gy for the tumor. Total RT doses were calculated by summation of each EQD2, external beam radiotherapy (EBRT), excluding the fractions with central shielding and ICBT, prescribed at point A. Regarding the status of persistent cervical cancer, we recorded tumor diameter, presence or absence of invasion into the parametrium or vaginal wall, the number of residual lymph node metastases and the period between completion of definitive RT/CCRT and diagnosis of persistent cervical cancer. Salvage HT was abstracted for mode of hysterectomy (simple hysterectomy, Piver-Rutledge type II or III radical hysterectomy), operation time, estimated blood loss and status of residual tumor at the end of surgery. Systemic CT was abstracted for the drugs to be used and response to the treatment assessed according to RECIST version 1.1. For survival outcomes, progression-free survival (PFS) and OS were recorded.

### Study definition

Cancer stage was re-classified based on the 2008 FIGO staging [16]. Definitive RT as the initial treatment consisted of whole-pelvis EBRT and/or high-dose rate ICBT. Patients who underwent interstitial brachytherapy were excluded. The methods for diagnosis of persistent cervical cancer were imaging tests (MRI, CT or PET) and histopathological examination of cervix. The confirmation of persistent tumor by histopathological examination was mandatory for any patient whose tumor was not detected by any imaging tests. PFS was the period from the start date of HT or salvage CT until the date of subsequent radiologic relapse or progression, or the date of last contact for disease-free patients. OS was the observed length of life from the start date of HT or salvage CT to death or the date of last contact. Early adverse events were defined as those that developed within 30 days after surgery, and late ones as those observed at least 30 days after surgery.

### Statistical analysis

We defined two treatment groups: a HT group and a CT group. The former comprised patients who underwent salvage HT for persistent cervical cancer and the latter included patients who received systemic CT. To control for confounders, the propensity score for selecting treatment was estimated by a multivariate logistic regression model, with treatment groups as dependent variables, and all the variables listed in Table 1 as covariates. Propensity score-matching was performed using a 1:1 matching with replacement, with a caliper width equal to 0.2 of the standard deviation of logit of propensity score, in which system subjects could be restored and extracted. Missing valuables were included in the model as separate categories; this is because missing data may systematically differ between the two treatment groups [17]. Log-rank test was carried out for survival analyses, and statistical significance was expressed with hazard ratio (HR) and 95% confidence interval (CI). Survival curves were constructed using the Kaplan-Meier method. *P* < 0.05 was considered statistically significant (all, two tailed). The cut-off value regarding complete resection of persistent cervical cancer in a HT group was evaluated using receiver operating characteristic (ROC) analysis. Statistical package for Social Science software (SPSS, version 12.0, Chicago, IL) and R (version 3.5.1, Vienna, Austria) were used for all the analyses.

**Table 1:** Baseline characteristics of patients treated with systemic chemotherapy versus salvage hysterectomy for persistent cervical cancer after definitive radiotherapy/concurrent chemoradiotherapy

	Original Data Set			Matched Data Set		
Variables	salvage HT	systemic CT	*P*	salvage HT	systemic CT	*P*
**Sample size, No.**	99	199		71	71	
**Variables at initial treatment**						
Histology						
SCC	70 (70.3)	142 (71.4)	0.766	52 (73.2)	52 (73.2)	1
ADC / ADSC	29 (29.3)	57 (28.1)		19 (26.8)	19 (26.8)	
Stage						
I	26 (26.3)	8 (4.0)	< 0.001	15 (21.1)	11 (15.5)	0.608
II	43 (43.4)	57 (28.6)		33 (46.5)	37 (52.1)	
III	29 (29.3)	107 (53.8)		22 (31.0)	23 (32.4)	
IV	1 (1.0)	27 (13.6)		1 (1.4)	0 (0)	
Tumor size, mm	54 (20–110)	57 (10–100)	0.085	55 (27–110)	50 (25–96)	0.238
Parametrial invasion						
yes	66 (67.3)	178 (90.4)	< 0.001	51 (71.8)	55 (77.5)	0.44
no	32 (32.7)	19 (9.6)		20 (28.2)	16 (22.5)	
Pelvic lymph node metastasis						
yes	43 (44.8)	119 (60.4)	0.015	32 (45.1)	40 (56.3)	0.735
no	53 (55.2)	78 (39.6)		39 (54.9)	31 (43.7)	
Para-aortic lymph node metastasis						
yes	8 (8.2)	21 (10.7)	0.506	7 (9.9)	13 (18.3)	0.148
no	89 (91.8)	175 (89.3)		64 (90.1)	58 (81.7)	
**Variables regarding definitive RT**						
IBCT						
yes	88 (88.9)	161 (80.9)	0.08	61 (85.9)	66 (93.0)	0.172
no	11 (11.1)	38 (19.1)		10 (14.1)	5 (7.0)	
Concurrent chemotherapy						
yes	89 (89.9)	166 (83.4)	0.134	66 (93.0)	65 (91.5)	0.754
no	10 (10.1)	33 (16.6)		5 (7.0)	6 (8.5)	
Boost irradiation						
yes	20 (20.2)	54 (27.3)	0.184	19 (26.8)	22 (31.0)	0.579
no	79 (79.8)	144 (72.7)		52 (73.2)	49 (69.0)	
^**a**^ **Variables at the diagnosis of persistent cervical cancer**						
Age, year	52 (26–82)	52 (26–81)	0.589	52 (26–82)	53 (26–78)	0.662
Performance status						
0	79 (83.2)	115 (62.8)	0.002	64 (90.1)	66 (93.0)	0.311
1	16 (16.8)	53 (29.0)		7 (7.0)	5 (7.0)	
2	0	10 (5.5)		0	0	
3	0	5 (2.7)		0	0	
Sites of persistent tumor						
cervix alone	72 (72.7)	97 (49.2)	< 0.001	20 (28.2)	23 (32.4)	0.468
^b^ cervix and the others	^c^ 27 (27.3)	100 (50.8)		^c^ 51 (71.8)	48 (67.6)	
Tumor size of persistent cervical disease, mm	21 (0–8)	30 (0–70)	0.011	22 (0–80)	30 (0–68)	0.156
0- ≤ 40 mm	70 (82.4)	106 (70.2)	0.055	58 (81.7)	57 (80.3)	1
> 40 mm	15 (17.6)	45 (29.8)		13 (18.3)	14 (19.7)	
Parametrial invasion of persistent tumor						
yes, and no extension to the pelvic wall	24 (25.8)	68 (42.0)	< 0.001	17 (23.9)	0 (0)	0.749
yes, and extension to the pelvic wall	1 (1.1)	33 (20.4)		1 (1.4)	22 (31.0)	
no parametrial invasion	68 (73.1)	61 (37.7)		53 (74.6)	49 (69.0)	
Vaginal invasion of persistent tumor						
yes	19 (20.0)	44 (26.7)	0.227	14 (19.7)	24 (33.8)	0.058
no	76 (80.0)	121 (73.3)		57 (80.3)	47 (66.2)	
Residual pelvic lymph node metastasis						
0	82 (82.8)	141 (72.3)	0.027	58 (81.7)	58 (81.7)	0.29
1	11 (11.1)	20 (10.3)		8 (11.3)	4 (5.6)	
2 or more	6 (6.1)	34 (17.4)		5 (7.0)	9 (12.7)	

Number (%), or median (range) is shown. The original data sets were not available for pelvic and para-aortic lymph node metastasis (n = 5) and parametrial invasion (*n* = 3) at initial treatment, boost irradiation (n = 1) for definitive RT, and performance status at the diagnosis of persistent cervical cancer (*n* = 20), sites of persistent tumor (n = 2), tumor size of persistent cervical disease (*n* = 62), parametrial invasion (*n* = 44) and vaginal invasion of persistent tumor (*n* = 38), and residual lymph node metastasis (*n* = 4)

Abbreviations: *HT* hysterectomy; *CT* chemotherapy; *SCC* squamous cell carcinoma; *ADC/ADSC* adenocarcinoma/adenosquamous cell carcinoma; *RT* radiotherapy; and *IBCT* intracavitary brachytherapy

^a^Diagnosis method of persistent tumor were imaging tests (MRI, CT or PET) and histopathological examination of cervix

^b^Patients with parametrial invasion, vaginal invasion, corpus invasion and persistent lymph node tumor were included in ‘cervix and the others’

^c^The number in the original data set is less than in the matched data set because subjects could be restored and extracted in the propensity score-matching system

## Results

### Demographics and propensity score matching

**Fig. 1:**
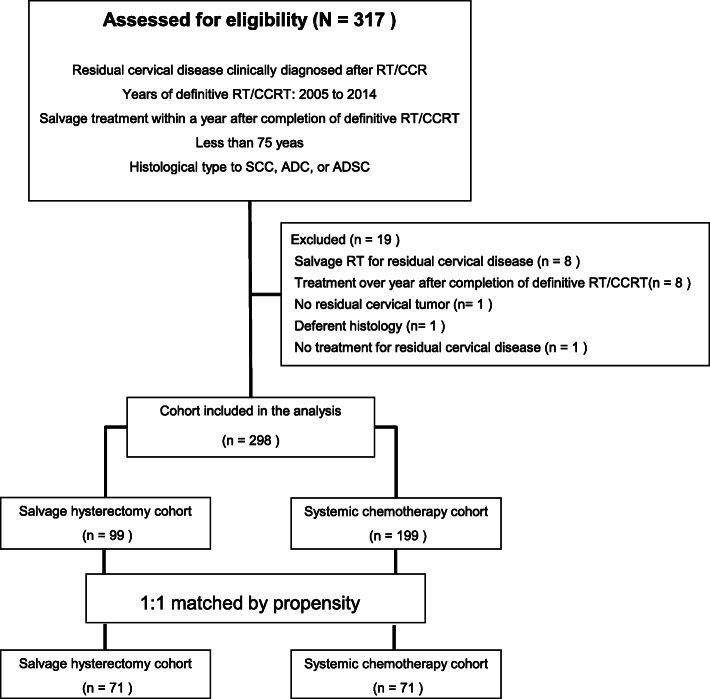
Study selection schema (*n* = 317). RT, radiotherapy; CCRT, concurrent chemoradiotherapy; SCC, squamous cell carcinoma; ADC, adenocarcinoma; ADSC, adenosquamous cell carcinoma

Between 2005 and 2014, we identified 309 patients from 35 institutions who underwent salvage HT or systemic CT; 298 patients were included in the analysis (Fig. 1). Among the original data set, and when compared to those who received systemic CT, patients who underwent salvage HT were more likely to have FIGO stage I-II disease and pelvic lymph node metastasis at the initial treatment, PS of 0 at the diagnosis of persistent tumor, small persistent cervical tumor with no parametrial invasion, and no other persistent disease besides that affecting the cervix (Table 1). A propensity match was then performed, and 142 patients were 1:1 matched, comprising a HT and CT cohort. The propensity score distributions for the cohorts both before and after matching are shown in Supplementary Figure A1; Figure A1A demonstrates an initial dissimilarity across the two cohorts on the basis of the propensity score distributions. However, after matching, the distributions of the propensity score for the two cohorts (Supplementary Figure. A1B) were quite homogenous and demonstrated the adequacy of the propensity score model to achieve balance. Table 1 shows that covariates after matching were well balanced with no significant differences in demographic or tumor-related variables between cohorts. The median EDQ2 of definitive RT/CCRT was 68.1 Gy (49.5–81.0) in the HT cohort and 64.0 Gy (35.4–74.0) in the CT cohort. The median time of diagnosis of persistent cervical cancer and of salvage HT or systemic CT after completion of definitive RT/CCRT were 51 days (0–276) and 98 days (13–343) in the HT cohort, and 50 days (0–230) and 96 days (13–265) in the CT cohort. The method of hysterectomy included 37 simple hysterectomies, 7 Piver-Rutledge type II radical hysterectomies, 23 Piver-Rutledge type III radical hysterectomies and 4 unknowns. Laparotomy was the procedure of choice for all surgeries. Out of 71 patients in the HT cohort, 20 received post-operative chemotherapy (18 taxane + platinum combination therapies, 2 other platinum therapies). In the CT cohort, 14 patients underwent hysterectomy after systemic CT (Supplementary Table A1).

### Perioperative adverse events in the HT cohort

The severe postoperative adverse events in the HT cohort, which were assessed by CTC AE version 4.0, are summarized in Table 2. Eleven late severe adverse events occurred in 7 patients (9.9%): 3 abscesses, 1 wound dehiscence, 1 ileus, 2 hydronephroses, 2 vesicovaginal fistulas, 1 rectovaginal fistula and 1 lymphocele, and 4 early severe adverse events developed in 3 patients (4.2%): 2 abscesses, 1 wound dehiscence and 1 lymphocele. With regard to intraoperative complications, there was one incidence of ureteral injury, which was fixed during surgery and did not lead to any adverse events after surgery. Nineteen patients underwent blood transfusion in the perioperative period. We have had no treatment-related deaths. We were unable to identify predictors of severe adverse events.

**Table 2:** Postoperative adverse events for salvage hysterectomy

	Early adverse events		Late adverse events	
Events (*n* = 71)	Any grade	Grade 3/4	Any grade	Grade 3/4
abscess	3^a^ (4.2)	2 (2.8)	3 (4.2)	3 (4.2)
wound dehiscence	1 (1.4)	1 (1.4)	1 (1.4)	1 (1.4)
vaginal cuff dehiscence	2 (2.8)	0 (0)	3^b^ (4.2)	0 (0)
ileus	1 (1.4)	0 (0)	1 (1.4)	1 (1.4)
hydronephrosis	3 ^a^ (4.2)	0 (0)	4^b^ (5.6)	2 (2.8)
vesicovaginal fistula	1^a^ (1.4)	0 (0)	3^b^ (4.2)	2 (2.8)
Rectovaginal fistula	1 (1.4)	0 (0)	3 (4.2)	1 (1.4)
lymphocele	3^a^ (4.2)	1 (1.4)	3^b^ (4.2)	1 (1.4)
lymph edema	15 (21.1)	0 (0)	14^b^ (19.7)	0 (0)

Number (%) is shown

^a^Early adverse events with unknown grade were included in abscess (*n* = 1), hydronephrosis (*n* = 2), vesicovaginal fistula (*n* = 1) and lymphocele (*n* = 1)

^b^Late adverse events with unknown grade were included in vaginal cuff dehiscence (*n* = 1), hydronephrosis (*n* = 1), vesicovaginal fistula (*n* = 1), lymphocele (*n* = 1) and lymph edema (*n* = 2)

### Survival analysis in the matched data set

**Fig. 2:**
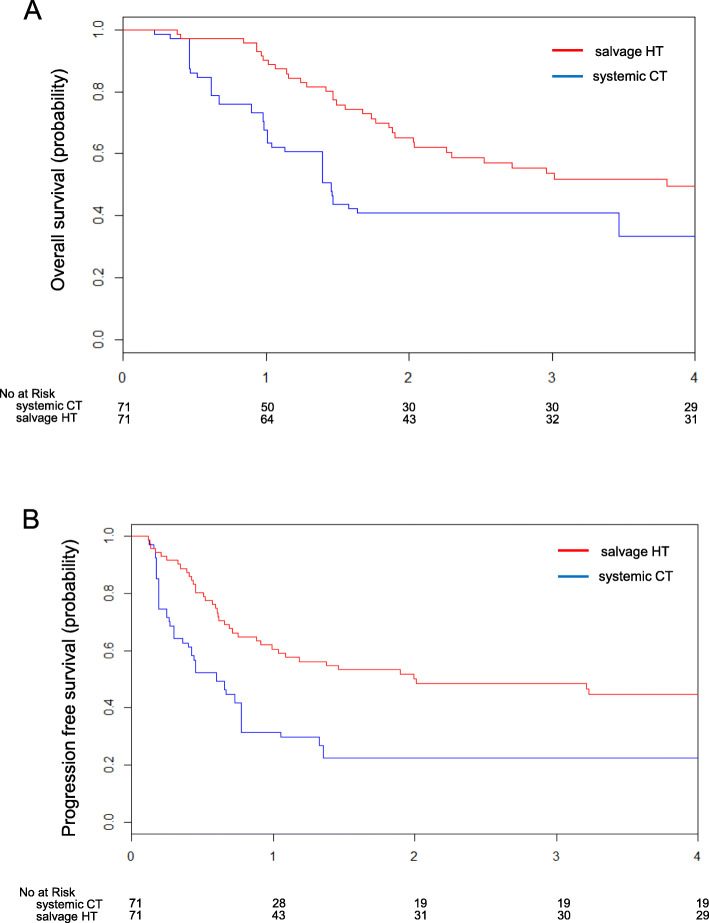
Propensity score matched survival outcomes of patients with persistent cervical cancer after RT/CCRT (systemic CT (*n* = 71) vs. salvage HT (*n* = 71)). **a** Kaplan-Meier curve of overall survival. **b** Kaplan-Meier curve of progression free survival. RT, radiotherapy; CCRT, concurrent chemoradiotherapy; systemic CT, systemic chemotherapy; salvage HT, salvage hysterectomy

The median follow-up period for OS was 4.6 years in the propensity score matched data set. Figure 2 shows OS and PFS estimated by the Kaplan-Meier method. OS for patients in the HT cohort was significantly longer than patients in the CT cohort (median OS, 3.8 years vs. 1.5 years, respectively; stratified log-rank *P* = 0.00193). Overall mortality was reduced in the HT cohort, with an estimated HR of 0.41 (95% CI, 0.23–0.73). PFS for patients in the HT cohort was also significantly longer than that of patients in a CT cohort (median PFS, 2.0 years vs. 0.7 years, respectively; stratified log-rank *P* = 0.00463). Overall risk of recurrence was reduced in the HT cohort, with an estimated HR of 0.45 (95% CI, 0.25–0.79).

### Relationship between the resectability of the residual cervical tumor for salvage hysterectomy and survival

A flow chart in supplementary Figure A2 shows distribution of patients in the HT cohort classified by status of residual tumor after hysterectomy. In the HT cohort, 67 out of 71 patients underwent resection with no macroscopic residual tumor (R0 group); microscopically complete resection was achieved in 10 of these patients. The remaining 4 patients in the HT cohort underwent resection with macroscopic tumor after surgery (R1 group) (refer to the footnote of supplementary Fig. A2, which provides the definition criteria for residual tumors).

**Fig. 3:**
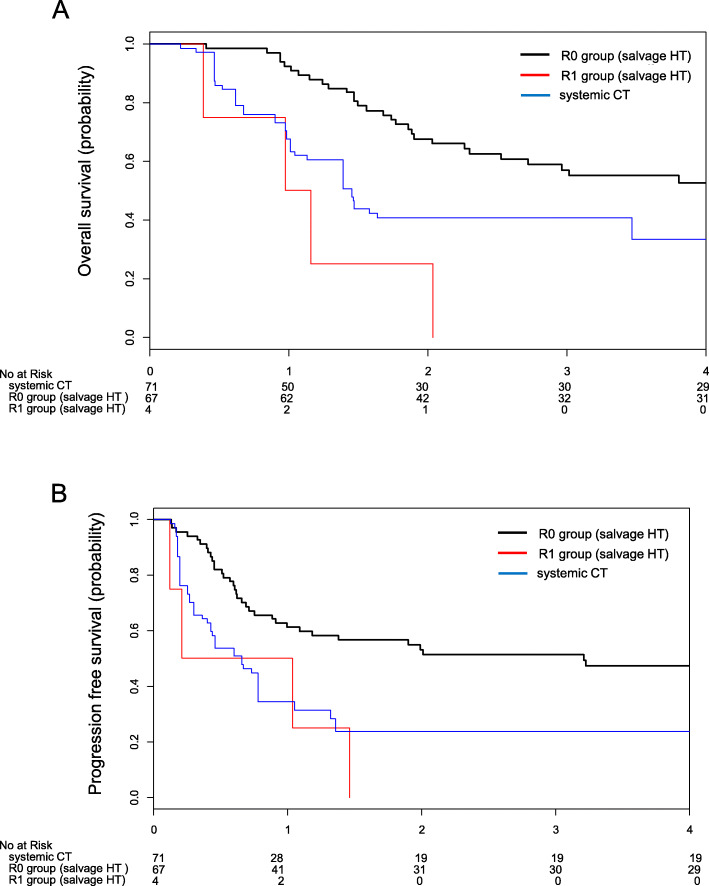
Survival outcomes of the R0 group (*n* = 67), R1 group (*n* = 4) and systemic CT (n = 71). **a** Kaplan-Meier curve of overall survival. **b** Kaplan-Meier curve of progression free survival. Systemic CT, systemic chemotherapy; R0 group, patients with no macroscopic residual tumor; R1 group, patients with macroscopic residual tumor after salvage hysterectomy

Patients in the R0 group had a significantly longer survival compared to those in the R1 group and those in the CT cohort (Fig. 3). Median OS was ‘not estimated’ in the R0 group, 1.0 year in the R1 group and 1.5 years in the CT cohort (Log-rank test, *P* < 0.001) (Fig. 3a). The HR of R1 with reference to R0 was ‘infinity’ (0.00–∞), and HR of CT with reference to R0 was 3.25 (1.702–6.207). Median PFS was 3.2 years in the R0 group, 0.2 years in the R1 group and 0.7 years in the CT cohort (Log-rank test, *P* = 0.001) (Fig. 3b). HR of R1 with reference to R0 was 8.761 (0.837–91.873), and HR of CT with reference to R0 was 2.973 (1.557–5.487). HR of CT with reference to R1 was 0.333 (0.035–3.205).

**Fig. 4:**
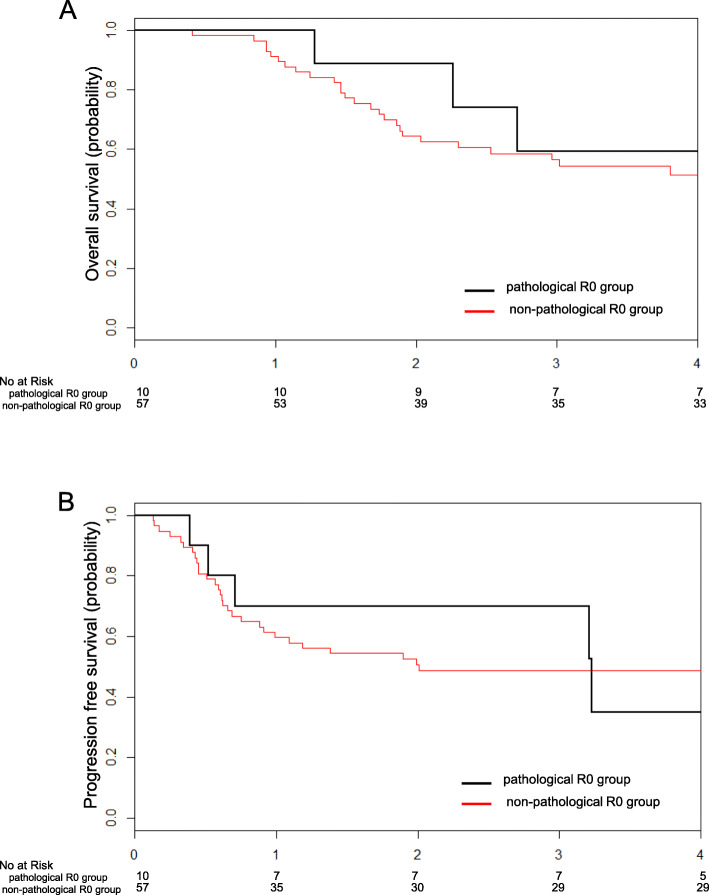
Survival outcomes of pathological R0 group (*n* = 10) and non-pathological R0 group (*n* = 57). **a** Kaplan-Meier curve of overall survival. **b** Kaplan-Meier curve of progression free survival. Pathological R0 group, patients who obtained complete resection macroscopically, and also microscopically; non-pathological R0 group, patients who had microscopically residual tumor, even though they had no macroscopic tumor after salvage hysterectomy

Out of 67 patients in the R0 group, 10 patients who obtained microscopically complete resection were grouped into the pathological R0 group and the remaining 57 were grouped into the non-pathological R0 group (refer to the footnote of supplementary Fig. A2, which provides the definition criteria for residual tumors). There was no significant survival difference between the pathological and non-pathological R0 groups (Fig. 4). Median OS of both groups was not estimated (Log-rank test, *P* = 0.500, HR 0.653, 0.198–2.158) (Fig. 4a). Median PFS was 3.2 years in the pathological R0 group and 2.0 years in the non-pathological R0 group (Log-rank test, *P* = 0.939, HR 0.947, 0.367–2.449) (Fig. 4b).

Predictive factors regarding R0 and pathological R0 resection were evaluated by logistic regression analysis using all variables in Table 1 in addition to the method of hysterectomy (simple hysterectomy, Piver-Rutledge type II or type III radical hysterectomy), corpus invasion of the persistent tumor (yes or no) and resection of lymph node (yes or no). On univariate analysis, corpus invasion of the persistent tumor (Odds ratio (OR) 8.17, 95% CI 0.80–83.66) and increasing tumor size (OR1.11, 95% CI 1.03–1.19) were independently associated with R0 resection. On multivariate analysis, increasing tumor size of persistent cervical cancer alone remained as significant predictive factor associated with high incomplete resection rate (*P* = 0.016, OR1.11, 95% CI 1.02–1.22). With reference to pathological R0, both uni and multivariate analysis revealed that increasing tumor size of persistent cervical cancer alone was significantly associated with a high incomplete pathological resection rate (*P* = 0.0385, OR1.06, 95% CI 1.003–1.126). The cut-off values of the persistent tumor diameter for R0 resection and pathological R0 resection after ROC analysis were 40 mm (sensitivity 100%, specificity 80.6%) and 11 mm (sensitivity 70%, specificity 86.9%), respectively (supplementary Figure. A3).

## Discussion

In this study on the effectiveness of salvage HT for patients with persistent cervical cancer after definitive RT/CCRT, we found the following two major points. First, the adoption of salvage HT reduced mortality rate up to about 60%, compared to systemic CT. Second, the relative resectability of a persistent tumor obviously affected survival, and tumor size alone was a significant predictive factor regarding resectability.

We showed that, when compared to systemic CT, salvage HT provided a significant PFS and OS improvement for patients with persistent cervical cancer after definitive RT/CCRT. These patients have an extremely poor prognosis due to their chemoresistance [5, 18, 19]. Therefore, our findings have important clinical implications, as they may provide a strategy to address an unmet medical need. Salvage HT has been suggested to impart benefits for treatment of persistent disease after RT since the 1990s [9–15]. However, all of the reports to support this conclusion were based on retrospective studies from single institutions with small sample sizes. Furthermore, no randomized trials have directly compared salvage HT and systemic CT, and thus the clinical role of surgical treatment has remained unclear. Specifically, there has been a lack of Level 1 evidence to guide management for patients with persistent tumors after RT/CCRT. To overcome the limitations of published noncomparative retrospective studies, we used propensity score matching with patients who underwent contemporary salvage HT to generate comparable subjects in a systemic CT group. To the best of our knowledge, this is the first study to employ propensity-matched analysis to look at survival benefits in this patient population.

Several studies have examined ‘completion HT/adjuvant HT’ after RT or CCRT [20–25]. While the concept behind this approach is similar to that of salvage HT, there are some key differences. For example, salvage HT is a treatment specifically for patients in whom tumors persist after definitive RT/CCRT, whereas completion HT/adjuvant HT can be given to all patients who undergo definitive RT/CCRT. A meta-analysis has shown that completion HT/adjuvant HT is not superior to conventional surveillance with regard to survival benefit [25]. Therefore, the selection of HT after definitive RT/CCRT requires a careful patient selection strategy in order for it to be an effective treatment modality. We showed that the resectability of persistent tumor obviously affected survival, and that tumor size alone is a significant predictive factor regarding resectability. Therefore, the goal of salvage HT should be R0 resection, and we suggest that stratification based on residual tumor size could be used to identify appropriate candidates for salvage HT. Indeed, the heterogeneity of residual tumor size might explain why surgical treatment has not been applied in clinical practice more successfully, even though there have been some reports of the benefits of surgery [9–15]. The opportunity to identify patients who would not benefit from a morbid surgery is a key consideration for patients who are deemed to have resectable disease. Based on our results, we suggest that patients with persistent cervical tumors less than 40 mm, which do not affect bladder and/or rectum, could be potential candidates for salvage HT. A more reliable cut-off value for complete resection would be 11 mm, although our results did not show a significant survival difference between pathological and non-pathological R0 groups. Houvenaeghel et al. showed relatively good results of 3-year and 5-year survival rates (64.9 and 55.6%, respectively) after curative surgery for patients with residual tumors of 20 mm or more [14]. However, Azria et al. reported that the therapeutic effect of HT was disappointing in a small series of 10 patients who developed residual disease of 20 mm or more after CCRT [13].

Intensity of RT is the most important factor that affects treatment outcomes of cervical cancer patients treated with RT/CCRT. In the current study, RT doses were evaluated as EQD2, which quantifies the biological effect of any RT method and also takes account of dose-per-fraction or dose-rate [26]**.** Our study is the first to take the treatment intensity of RT into account when evaluating the efficacy of surgical treatment for cervical cancer after definitive RT. Indeed, previous studies, including prospective trials or meta-analysis, did not contain details of RT intensity [20–25, 27, 28], which might preclude the adoption of salvage HT after definitive RT/CCRT in the clinic. In the current study, the median dose of EQD2 in the HT cohort was 68.1 Gy, and this dose would ensure that RT/CCRT leads to a complete cure. Thus, we have shown that salvage HT could be a valuable curative intervention for persistent cervical tumors, even after definitively invasive treatment has been performed.

PE could be a curative option for some of those patients [6, 29]. Total PE offers a 5-year survival of 23–50%, however, high rate of severe postoperative complications, such as infection, injury of the urinary and gastrointestinal tracts, and small-bowel obstruction, occurs, in addition to a 4–14% surgery-related mortality rate [30–33]. The major clinical issue associated with surgical treatment after RT/CCRT is the high incidence of post-operative complications, and this must be balanced against the potential benefit of treatment. In this sense, PE has been employed sparingly. Therefore, salvage HT could be an acceptable alternative to PE and vaginectomy might be also allowed as salvage therapy for patients with recurrent vaginal tumors, in case that they had no prior pelvic radiotherapy [34]. The current study, in which patients who underwent PE were excluded, showed that early and late severe adverse events occurred in 4.2 and 9.9% of patients, respectively (Table 2). In a recent retrospective analysis of 362 locally advanced cervical cancer patients undergoing adjuvant HT after CCRT, grade 3 or 4 post-operative complications occurred in 5.8% of cases [35].

The current study has some limitations that should be taken into account when interpreting our results. First, there were some unbalanced variables present in the data, although these variables were not statistically significant between the two groups, even after propensity score matching was performed (Table 1). This may be explained by significant differences in the characteristics of treated populations in clinical practice, which would lead to large biases between the groups (supplementary Figure. A1A). The retrospective nature of the study meant that our ability to control for differences was limited to variables for which data were available. In spite of this limitation, we suggest that our current results, and our interpretation of them, are valid, and provide important information. Second, the salvage HT was not based on random assignment, and we could not control the eligibility criteria for salvage HT in the study. The result of the current study may be confounded by other unobserved variables. Although we used a rigorous statistical method to adjust for baseline differences between treatment groups, including propensity score matching, unobserved variables could be unbalanced across treatment groups and partly explain the difference in survival. Furthermore, safety records of the salvage HT could be changed depending on the eligibility criteria. Despite the recent development of vessel-sealing and cutting devices and advances in surgical techniques, salvage HT in a previously irradiated field is usually challenging, even in patients with seemingly resectable disease. Third, we were unable to determine the specific time at which the diagnosis of persistent tumor was made based on the available data. In the current study, the median time at which diagnosis was made in the salvage HT and systemic CT groups was 51 days and 50 days after completion of RT/CCRT, respectively. Some tumors continue to regress several months after radiation treatment, meaning that a complete response may eventually be achieved, even if the tumor existed at the time when RT was terminated. This selection bias is fully acknowledged. The opportunity to identify the appropriate time of diagnosis and understand tumor biology could make salvage HT a more appealing treatment strategy. Fourth, although systemic CT with bevacizumab significantly improves the survival for patients with advanced and recurrent cervical cancer [36], we could not compare its efficacy with that of salvage HT. This is because the current study included cases between 2005 and 2014, which is before bevacizumab was used for the treatment of cervical cancer. It is therefore still possible that bevacizumab, rather than salvage HT, could address the unmet medical need of patients with residual disease after definitive RT/CCRT.

## Conclusions

We have shown that the adoption of salvage HT for patients with persistent cervical cancer after definitive RT/CCRT significantly reduces mortality rate when compared to systemic CT. Furthermore, complete resection obviously affected survival compared to incomplete resection and systemic CT, and increased size of the persistent tumor is negatively correlated with successful resection rate. Further prospective clinical trials with regard to salvage HT after RT/CCRT are now warranted. The other survey to address appropriate patients for salvage HT after definitive RT/CCRT was conducted and several factors were identified as selection criteria for salvage HT (no publication). Based on the results of that study, the Japanese Clinical Oncology Group is now planning to conduct a multicenter prospective trial to address this issue.

## Supplementary Information

**Additional file 1 **: **Figure A1**. Propensity score distributions (A) before matching, and (B) after matching. HT, hysterectomy; CT, chemotherapy. **Figure A1A** demonstrates an initial dissimilarity across the two cohorts on the basis of the propensity score distributions. However, after matching, the distributions of the propensity score for the two cohorts (supplementary Figure A1B) were quite homogenous. This demonstrates that the propensity score model was able to achieve balance. Note: Propensity score-matching was performed using a 1:1 matching with replacement, with a caliper width equal to 0.2 of the standard deviation of logit of propensity. In this matching system, some subjects could be restored and extracted. **Figure A2**. Distribution of patients in the HT cohort classified by residual tumor status after hysterectomy. R0 group, patients with no macroscopic residual tumor; R1 group, patients with macroscopic residual tumor after salvage hysterectomy; pathological R0 group, patients in whom complete macroscopic or microscopic resection was obtained; non-pathological R0 group, patients with no macroscopic tumor after salvage hysterectomy, but in whom microscopic residual tumors could be detected.**Figure A3.** ROC analysis indicated that the cut-off values of the diameter for persistent tumors were 40 mm for R0 resection (A) and 11 mm for pathological R0 resection (B). **Figure A4**. Survival comparison using propensity matched data between salvage HT and systemic CT for patients with persistent cervical cancer after RT/CCRT. Patients with stage IB1 and IVA malignancies were excluded from the analysis. **Figure A5**. Survival comparison using propensity matched data between salvage HT and systemic CT for patients with persistent cervical cancer after RT/CCRT. Patients who did not undergo intracavity brachytherapy were excluded from the analysis. **Figure A6**. Survival comparison using propensity matched data between salvage HT and systemic CT for patients with persistent cervical cancer after RT/CCRT. Patients who had experienced crossover treatments were excluded from the analysis. **Figure A7**. Survival comparison using propensity matched data between salvage HT and systemic CT. Patients were divide into two groups according to whether imaging after definitive RT/CCRT revealed a residual lymph node tumor or not. **Figure A8.** Survival comparison using propensity matched data between salvage HT and systemic CT. Patients were divide into two groups according to whether imaging after definitive RT/CCRT revealed a residual cervical cancer or not. **Figure A9**. Survival comparison of patients in the systemic CT group, which was subdivided into 3 groups according to their response of each patient to chemotherapy (data are from the original data set). **Table A1**. Summary of treatments received. **Table A2**. Adverse events for systemic chemotherapy. **Table A3.** Evaluation of CT, MRI and PET-CT for persistent tumors after definitive RT/CCRT. **Table A4**. Sites of progressive disease after salvage HT or systemic CT. **Table A5.** Participating institutions and sample size.

## Data Availability

All relevant data are within the paper. The dataset supporting the conclusions of this article is available from the corresponding author on reasonable request.

## Abbreviations

AC: Adenocarcinoma
ASC: Adeno-squamous cell carcinoma
CCRT: Concurrent chemoradiotherapy
CT: Chemotherapy
CI: Confidence interval
EDQ2: Equivalent total doses in 2 Gy per fraction
EBRT: External beam radiotherapy
FIGO: International Federation of Gynecology and Obstetrics
HR: Hazard ratio
HPV: Human papilloma virus
HT: Hysterectomy
ICBT: Intracavitary brachytherapy
JCOG: the Japan Clinical Oncology Group
OR: Odds ratio
OS: Overall survival
PE: Pelvic exenteration
PFS: Progression-free survival
PS: Performance status
ROC: Receiver operating characteristic
RT: Radiotherapy
SCC: Squamous cell carcinoma

## References

[1] Siegel R, Miller KD, Jemal A (2017). Cancer statistics, 2017. CA Cancer J Clin.

[2] Chemoradiotherapy for Cervical Cancer Meta-Analysis Collaboration (2008). Reducing uncertainties about the effects of chemoradiotherapy for cervical cancer: a systematic review and meta-analysis of individual patient data from 18 randomized trials. J Clin Oncol.

[3] Eifel PJ, Winter K, Morris M, Levenback C, Grigsby PW, Cooper J (2004). Pelvic irradiation with concurrent chemotherapy versus pelvic and Para-aortic irradiation for high-risk cervical cancer: an update of radiation therapy oncology group trial (RTOG) 90-01. J Clin Oncol.

[4] Toita T, Kitagawa R, Hamano T, Umayahara K, Hirashima Y, Aoki Y (2012). Phase II study of concurrent chemoradiotherapy with high-dose-rate intracavity brachytherapy in patients with locally advanced uterine cervical cancer. Efficacy and toxicity of a low cumulative radiation schedule. Gynecol Oncol.

[5] Moore DH, Tian C, Monk BJ, Long HJ, Omura GA, Bloss JD (2010). Prognostic factors for response to cisplatin-based chemotherapy in advanced cervical carcinoma; a gynecologic oncology group study. Gynecol Oncol.

[6] NCCN (2020). Clinical Guidelines in Oncology: Cervical Cancer Version 1.

[7] Beckmann MW, Mallmann P (2009). Uterus Commission of the Gynecological Oncological Oncology Working Group (AGO). Interdisciplinary S2k guideline on the diagnosis and treatment of cervical carcinoma. J Cancer Res Clin Oncol.

[8] Nagase S, Inoue Y, Umesaki N, Aoki D, Ueda M, Sakamoto H (2010). Evidence-based guidelines for treatment of cervical cancer in Japan: Japan Society of Gynecologic Oncology (JSGO) 2007 edition. Int J Clin Oncol.

[9] Ota T, Takeshima N, Tabata T, Hasumi K, Takizawa K (2008). Adjuvant hysterectomy for treatment of residual disease in patients with cervical cancer treated with radiation therapy. Br J Cancer.

[10] Boers A, Arts HJ, Klip H, Nijhuis ER, Pras E, Hollema H (2014). Radical surgery in patients with residual disease after (chemo) radiation for cervical cancer. Int J Gynecol Cancer.

[11] Morice P, Uzan C, Zafrani T, Delpech Y, Gouy S, Haie-Meder C (2007). The role of surgery after chemoradiation therapy and brachytherapy for stage IB2/II cervical cancer. Gynecol Oncol.

[12] Touboul C, Uzan C, Mauguen A, Gouy S, Rey A, Pautier P (2010). Prognostic factors and morbidities after completion surgery in patients undergoing initial chemoradiation therapy for locally advanced cervical cancer. Oncologist.

[13] Azria E, Morice P, Haie-Meder C, Thoury A, Pautier P, Lhomme C (2005). Results of hysterectomy in patients with bulky residual disease at the end of chemoradiotherapy for stage IB2/II cervical carcinoma. Ann Surg Oncol.

[14] Houvenaeghel G, Lelievre L, Buttarelli M, Jacquemier J, Carcopino x VP (2007). Contribution of surgery in patients with bulky residual disease after chemoradiation for advanced cervical carcinoma. Eur J Surg Oncol.

[15] Gosset M, Chargari C, Bentivegna E, Leary A, Genestie C, Maulard A (2019). Should we cease to perform salvage hysterectomy after chemoradiation and brachytherapy in locally advanced cervical cancer?. Anticancer Res.

[16] Pecorelli S (2009). Revised FIGO staging for carcinoma of the vulva, cervix, and endometrium. Int J Gynaecol Obstet.

[17] Mattei A (2009). Estimating and using propensity score in presence of missing background data: an application to assess the impact of childbearing on wellbeing. Stat Methods Appl.

[18] Moore DH, Blessing JA, McQuellon RP, Thaler HT, Cella D, Benda J (2004). Phase III study of cisplatin with or without paclitaxel in stage IVB, recurrent, or persistent squamous cell carcinoma of the cervix: a gynecologic oncology group study. J Clin Oncol.

[19] Long HJ, Bundy BN, Grendys EC, Benda JA, McMeekin DS, Sorosky J (2005). Randomized phase III trial of cisplatin with or without topotecan in carcinoma of the uterine cervix: a gynecologic oncology group study. J Clin Oncol.

[20] Keys HM, Bundy BN, Stehman FB, Okagaki T, Gallup DG, Burnettet AF (2003). Radiation therapy with and without extrafascial hysterectomy for bulky stage IB cervical carcinoma: a randomized trial of the Gynecologic Oncology Group. Gynecol Oncol.

[21] Motton S, Houvenaeghel G, Delannes M, Querleu D, Soulé-Tholy M, Hoff J (2010). Results of surgery after concurrent chemoradiotherapy in advanced cervical cancer: comparison of extended hysterectomy and extrafascial hysterectomy. Int J Gynecol Cancer.

[22] Yang J, Yang J, Cao D, Shen K, Ma J, Zhang F (2020). Completion hysterectomy after chemoradiotherapy for locally advanced adeno-type cervical carcinoma: updated survival outcomes and experience in post radiation surgery. J Gynecol Oncol.

[23] Fanfani F, Vizza E, Landoni F, Iaco P, Ferrandina G, Corrado G (2016). Radical hysterectomy after chemoradiation in FIGO stage III cervical cancer patients versus chemoradiation and brachytherapy: complications and 3-years survival. Eur J Surg Oncol.

[24] Legge F, Chiantera V, Macchia G, Fagotti A, Fanfani F, Ercoli A (2015). Clinical outcome of recurrent locally advanced cervical cancer (LACC) submitted to primary multimodality therapies. Gynecol Oncol.

[25] Shim SH, Kim SN, Chae SH, Kim JE, Lee SJ (2018). Impact of adjuvant hysterectomy on prognosis in patients with locally advanced cervical cancer treated with concurrent chemoradiotherapy: a meta-analysis. J Gynecol Oncol.

[26] Fowler JF (1989). The linear quadratic formula and progress in fractionated radiotherapy. Br J Radiol.

[27] Morice P, Rouanet P, Rey A, Romestaing P, Houvenaeghel G, Boulanger JC (2012). Results of the GYNECO 02 study, an FNCLCC phase III trial comparing hysterectomy with no hysterectomy in patients with a (clinical and radiological) complete response after chemoradiation therapy for stage IB2 or II cervical cancer. Oncologist.

[28] Cetina L, Gonzalez-Enciso A, Cantu D, Coronel J, Pérez-Montiel D, Hinojosaet J (2013). Brachytherapy versus radical hysterectomy after external beam chemoradiation with gemcitabine plus cisplatin: a randomized phase III study in IB2-IIB cervical cancer patients. Ann Oncol.

[29] Stanhope CR, Webb MJ, Podratz KC (1990). Pelvic exenteration for recurrent cervical cancer. Clin Obstet Gynecol.

[30] Rutledge FN, Smith JP, Wharton JT, O’Quinn AG (1977). Pelvic exenteration: analysis of 296 patients. Am J Obstet Gynecol.

[31] Shingleton HM, Soong SJ, Gelder MS, Hatch KD, Baker VV, Austin JM (1989). Clinical and histopathologic factors predicting recurrence and survival after pelvic exenteration for cancer of the cervix. Obstet Gynecol.

[32] Morley GW, Hopkins MP, Lindenauer SM, Roberts JA (1989). Pelvic exenteration, University of Michigan: 100 patients at 5 years. Obstet Gynecol.

[33] Moutardier V, Houvenaeghel G, Martino M, Lelong B, Bardou VJ, Resbeut M (2004). Surgical resection of locally cervical cancer: a single institutional 70 patients series. Int J Gynecol Cancer.

[34] Vizzielli G, Tortorella L, Conte C, Chiantera V, Gallotta V, Foschi N, et al. Is a Vaginectomy enough or is a pelvic Exenteration always required for surgical treatment of recurrent cervical Cancer? A Propensity-Matched Study. Ann Surg Oncol. 2020. 10.1245/s10434-020-09207-w. 10.1245/s10434-020-09207-w 33063258

[35] Ferrandina G, Ercoli A, Fatotti A, Fanfani F, Gallotta V, Margariti AP (2014). Completion surgery after concomitant chemoradiation in locally advanced cervical cancer: a comprehensive analysis of pattern of postoperative complications. Ann Surg Oncol.

[36] Tewari KS, Sill MW, Long HJ, Penson RT, Huang H, Ramondetta LM (2014). Improved survival with bevacizumab in advanced cervical cancer. N Engl J Med.

