# Supplementary material for: Propensity score-matched analysis of systemic chemotherapy versus salvage hysterectomy for persistent cervical cancer after definitive radiotherapy/concurrent chemoradiotherapy
Source: BMC Cancer. 2020 Nov 30;20:1169. doi: 10.1186/s12885-020-07672-w (PMC7708164; doi:10.1186/s12885-020-07672-w)
Supplement: Supplementary file 1 — Additional file 1 : Figure A1. Propensity score distributions (A) before matching, and (B) after matching. HT, hysterectomy; CT, chemotherapy. Figure A1A demonstrates an initial dissimilarity across the two cohorts on the basis of the propensity score distributions. However, after matching, the distributions of the propensity score for the two cohorts (supplementary Figure A1B) were quite homogenous. This demonstrates that the propensity score model was able to achieve balance. Note: Propensity score-matching was performed using a 1:1 matching with replacement, with a caliper width equal to 0.2 of the standard deviation of logit of propensity. In this matching system, some subjects could be restored and extracted. Figure A2. Distribution of patients in the HT cohort classified by residual tumor status after hysterectomy. R0 group, patients with no macroscopic residual tumor; R1 group, patients with macroscopic residual tumor after salvage hysterectomy; pathological R0 group, patients in whom complete macroscopic or microscopic resection was obtained; non-pathological R0 group, patients with no macroscopic tumor after salvage hysterectomy, but in whom microscopic residual tumors could be detected.Figure A3. ROC analysis indicated that the cut-off values of the diameter for persistent tumors were 40 mm for R0 resection (A) and 11 mm for pathological R0 resection (B). Figure A4. Survival comparison using propensity matched data between salvage HT and systemic CT for patients with persistent cervical cancer after RT/CCRT. Patients with stage IB1 and IVA malignancies were excluded from the analysis. Figure A5. Survival comparison using propensity matched data between salvage HT and systemic CT for patients with persistent cervical cancer after RT/CCRT. Patients who did not undergo intracavity brachytherapy were excluded from the analysis. Figure A6. Survival comparison using propensity matched data between salvage HT and systemic CT for patients with persistent cervical [file 12885_2020_7672_MOESM1_ESM.docx]

Figure. A1. Propensity score distributions (A) before matching, and (B) after matching. HT, hysterectomy; CT, chemotherapy.


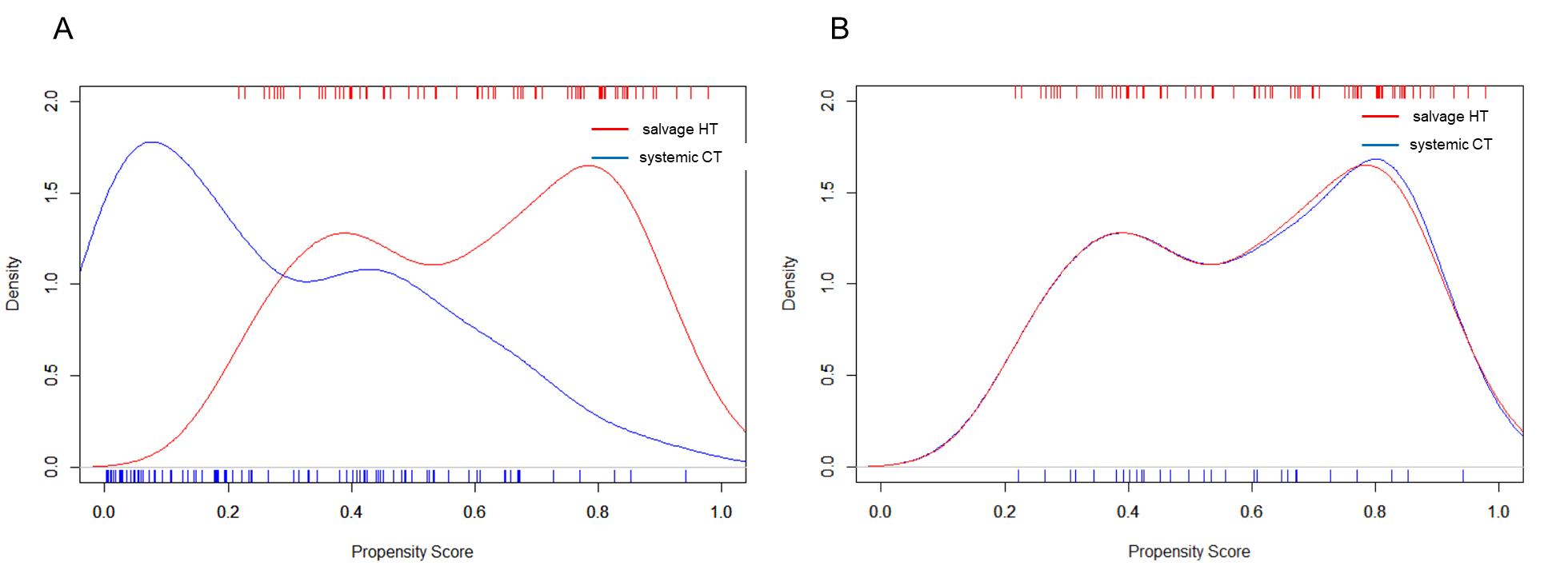


Figure A1A demonstrates an initial dissimilarity across the two cohorts on the basis of the propensity score distributions. However, after matching, the distributions of the propensity score for the two cohorts (supplementary Figure A1B) were quite homogenous. This demonstrates that the propensity score model was able to achieve balance.

Note: Propensity score-matching was performed using a 1:1 matching with replacement, with a caliper width equal to 0.2 of the standard deviation of logit of propensity. In this matching system, some subjects could be restored and extracted.

Figure. A2. Distribution of patients in the HT cohort classified by residual tumor status after hysterectomy.


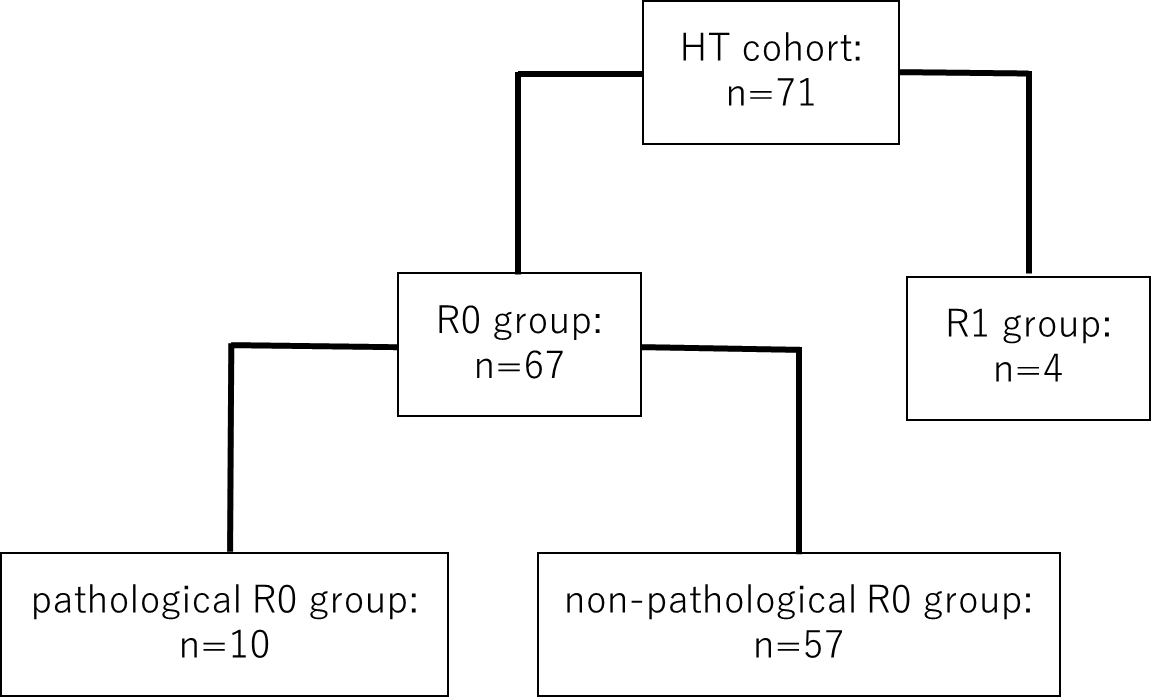


R0 group, patients with no macroscopic residual tumor; R1 group, patients with macroscopic residual tumor after salvage hysterectomy; pathological R0 group, patients in whom complete macroscopic or microscopic resection was obtained; non-pathological R0 group, patients with no macroscopic tumor after salvage hysterectomy, but in whom microscopic residual tumors could be detected.

Figure. A3. ROC analysis indicated that the cut-off values of the diameter for persistent tumors were 40 mm for R0 resection (A) and 11 mm for pathological R0 resection (B).


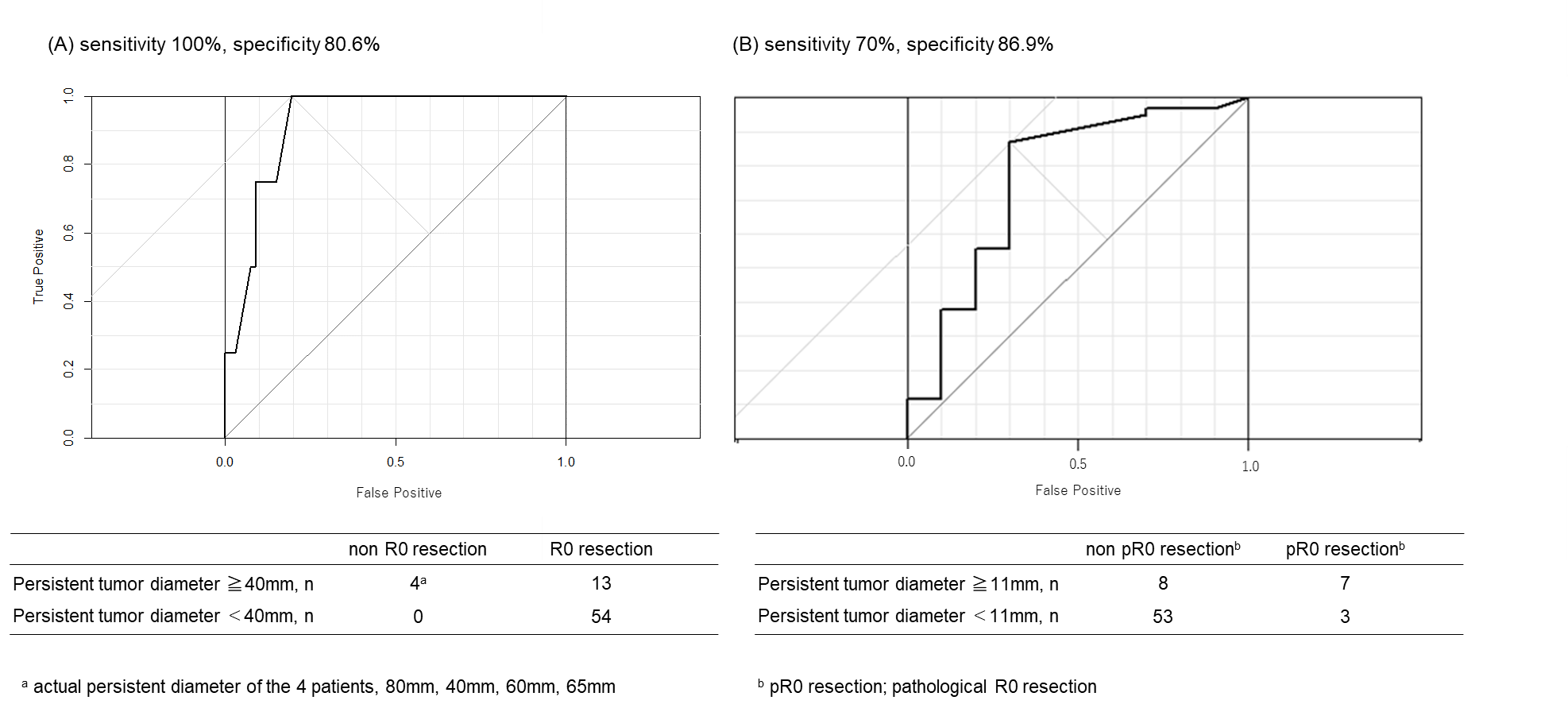


Figure A4. Survival comparison using propensity matched data between salvage HT and systemic CT for patients with persistent cervical cancer after RT/CCRT. Patients with stage IB1 and IVA malignancies were excluded from the analysis.


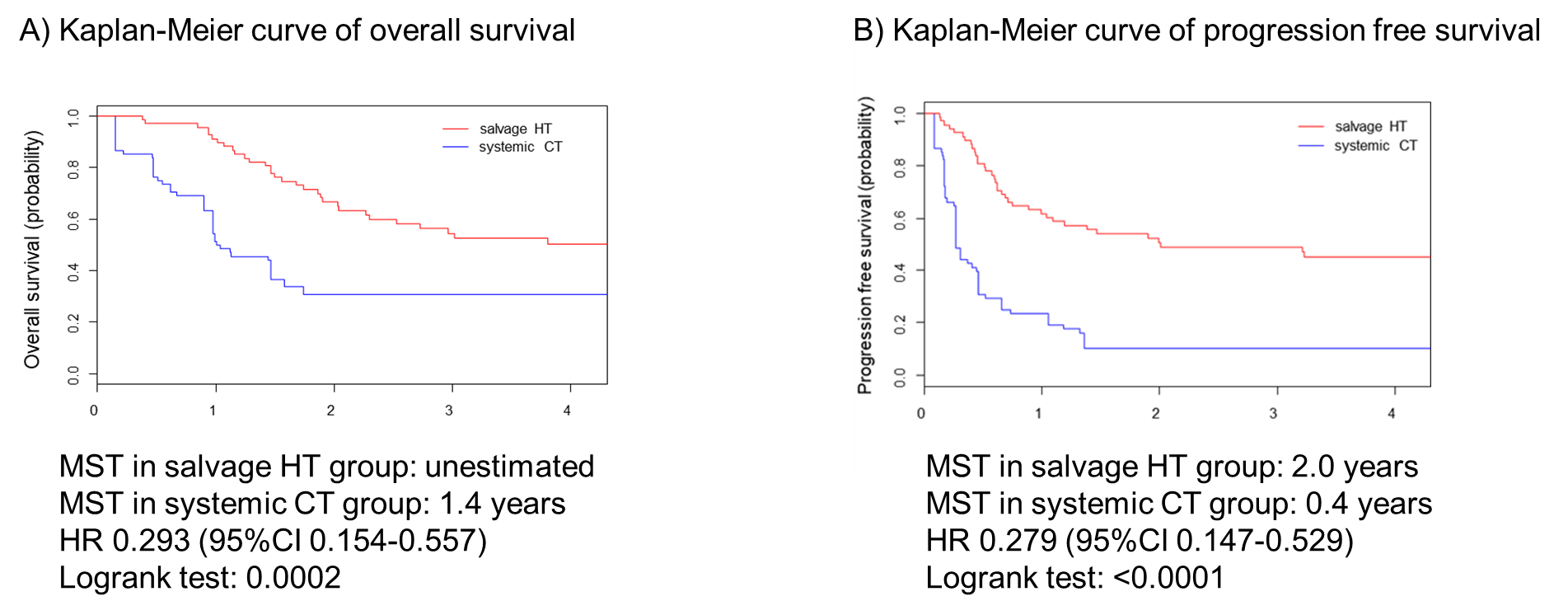


Figure A5. Survival comparison using propensity matched data between salvage HT and systemic CT for patients with persistent cervical cancer after RT/CCRT. Patients who did not undergo intracavity brachytherapy were excluded from the analysis.


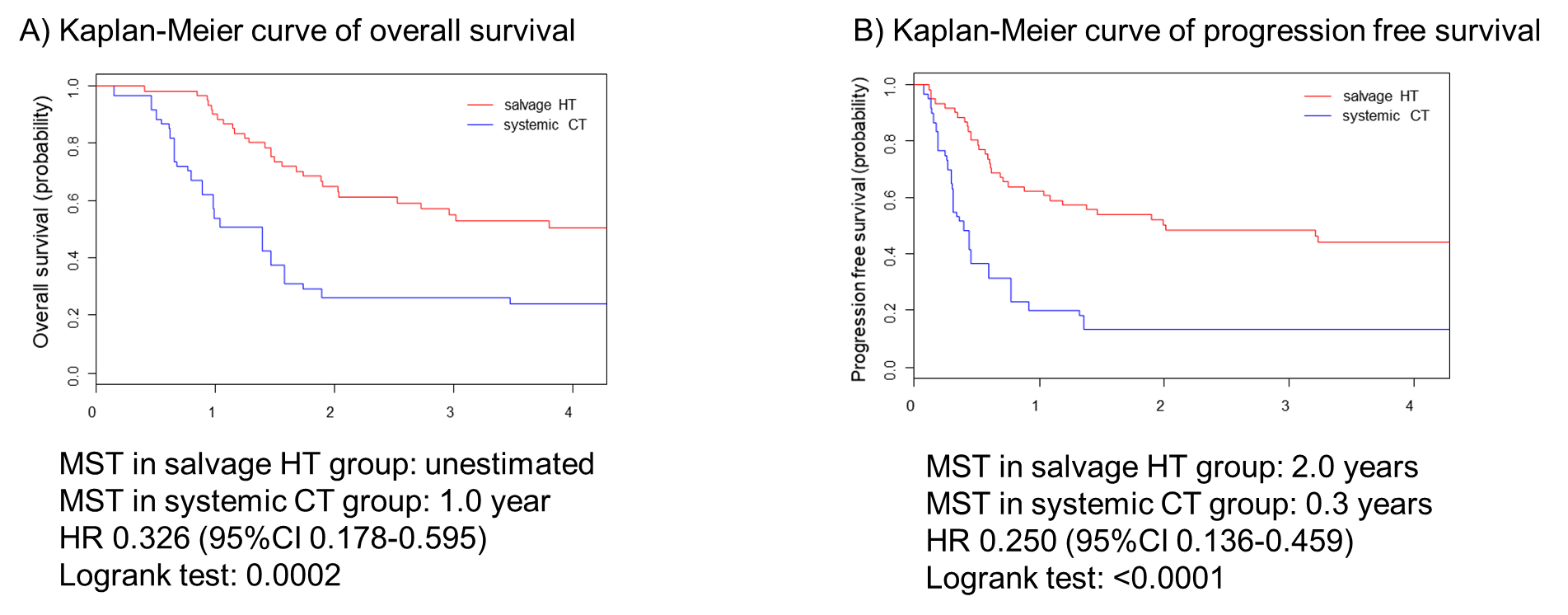


Figure A6. Survival comparison using propensity matched data between salvage HT and systemic CT for patients with persistent cervical cancer after RT/CCRT. Patients who had experienced crossover treatments were excluded from the analysis.


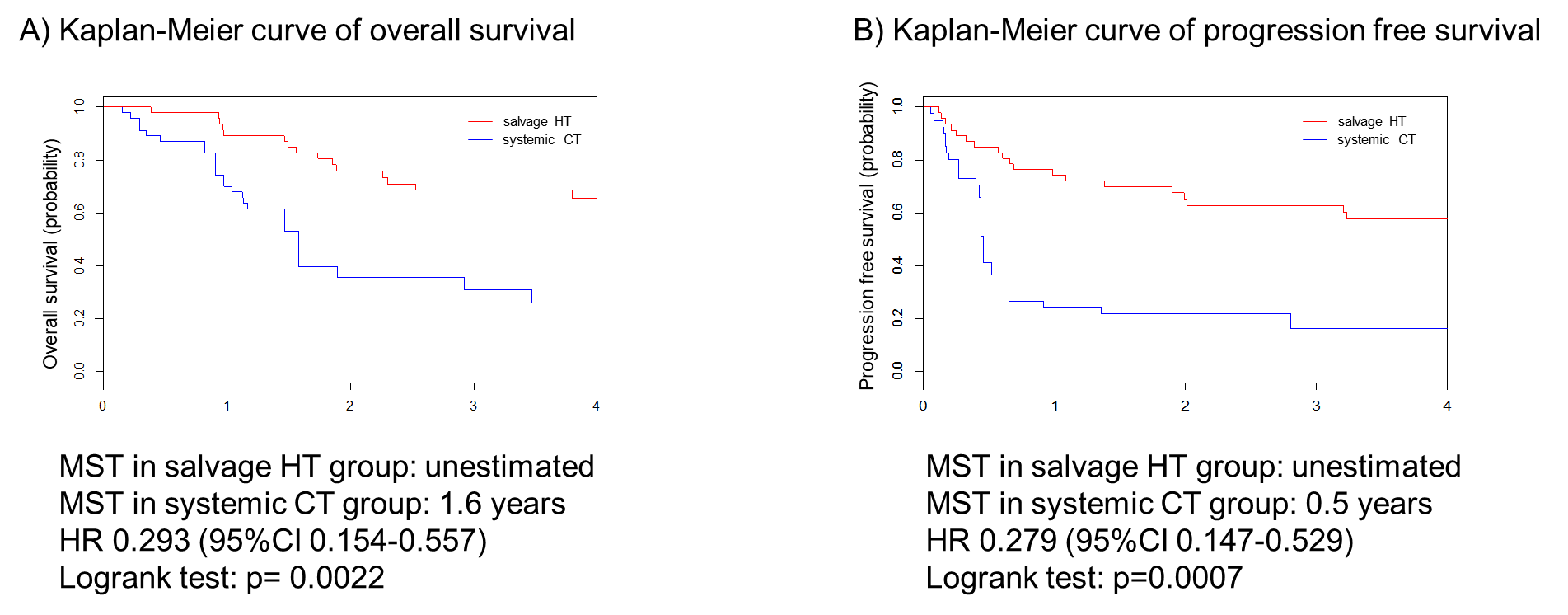


Figure A7. Survival comparison using propensity matched data between salvage HT and systemic CT. Patients were divide into two groups according to whether imaging after definitive RT/CCRT revealed a residual lymph node tumor or not.

**Patients with no apparent residual lymph-node tumor upon imaging**


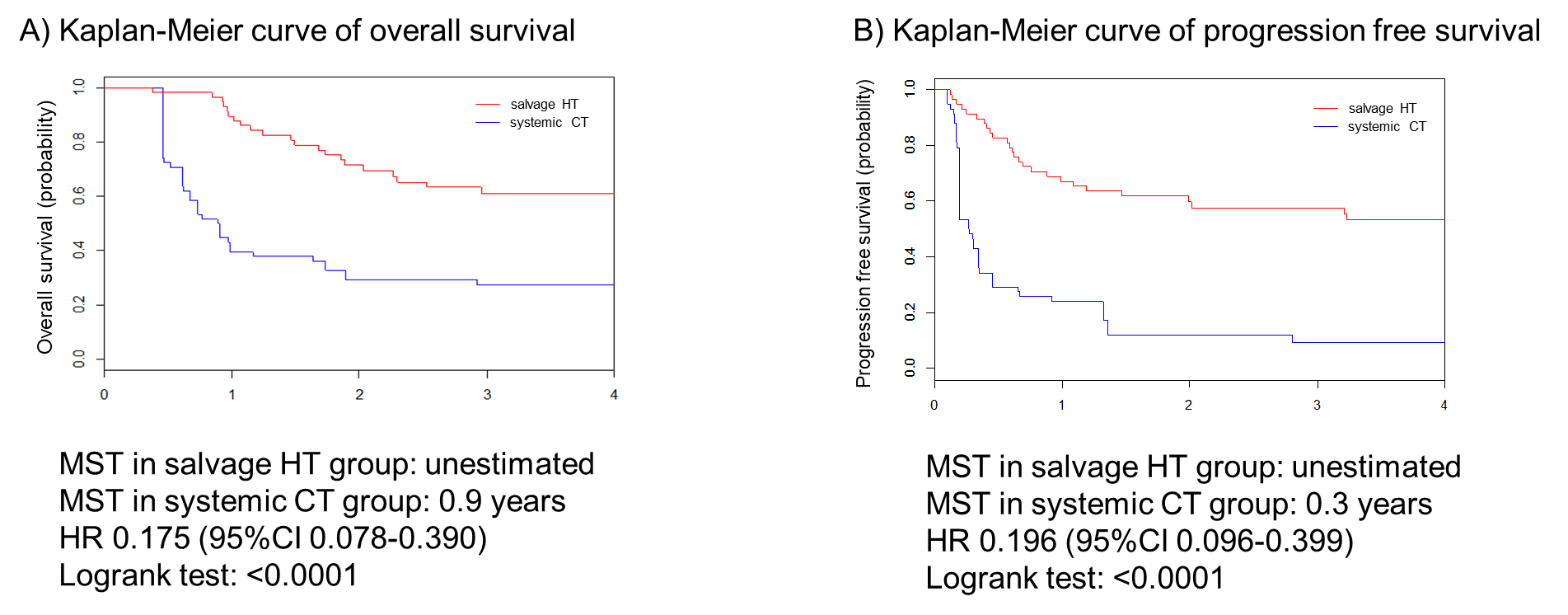


**Patients with an apparent residual lymph-node tumor upon imaging**

Not estimated:

The propensity score could not be estimated because a complete separation occurred. The number of subjects with an apparent residual lymph-node tumor was too low to perform a meaningful analysis.

Figure A8. Survival comparison using propensity matched data between salvage HT and systemic CT. Patients were divide into two groups according to whether imaging after definitive RT/CCRT revealed a residual cervical cancer or not.

**Patients with no apparent residual cervical cancer upon imaging**

Not estimated:

The propensity score could not be estimated because a complete separation occurred. The number of subjects with an apparent residual cervical cancer was too low to perform a meaningful analysis.

**Patients with an apparent residual cervical cancer upon imaging**


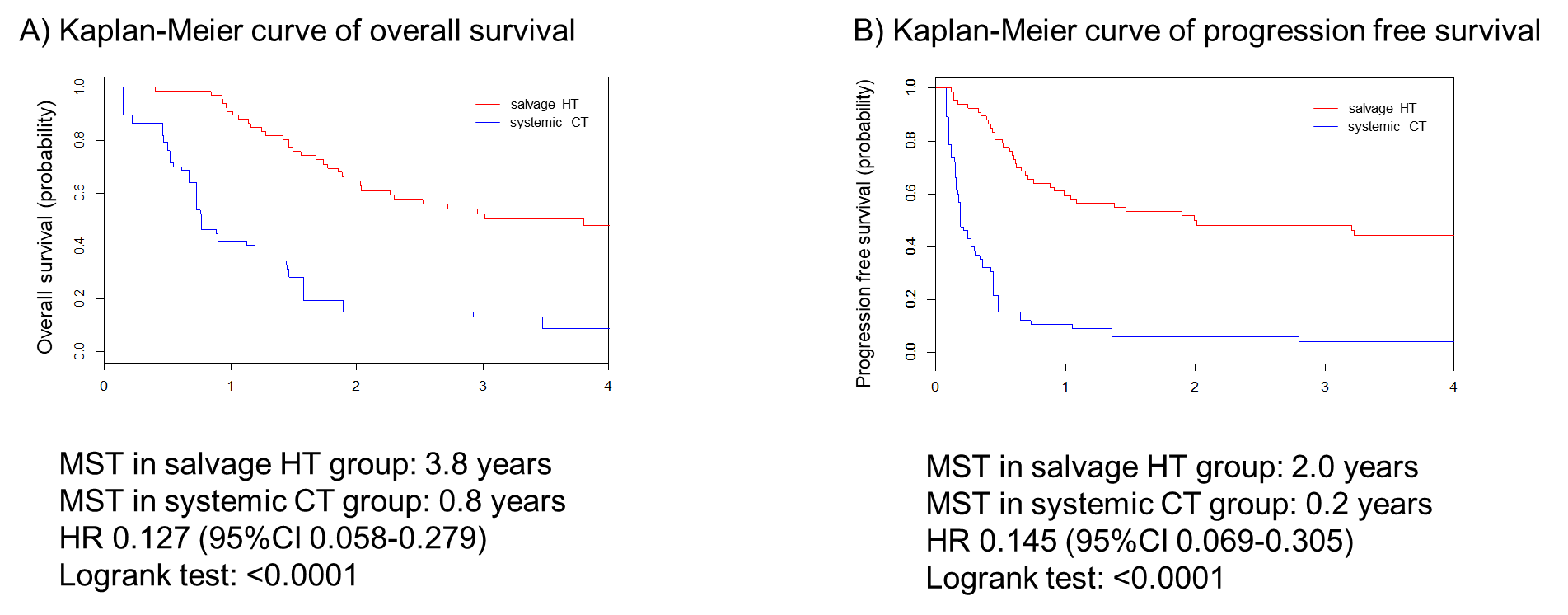


Figure A9.

Survival comparison of patients in the systemic CT group, which was subdivided into 3 groups according to their response of each patient to chemotherapy (data are from the original data set).


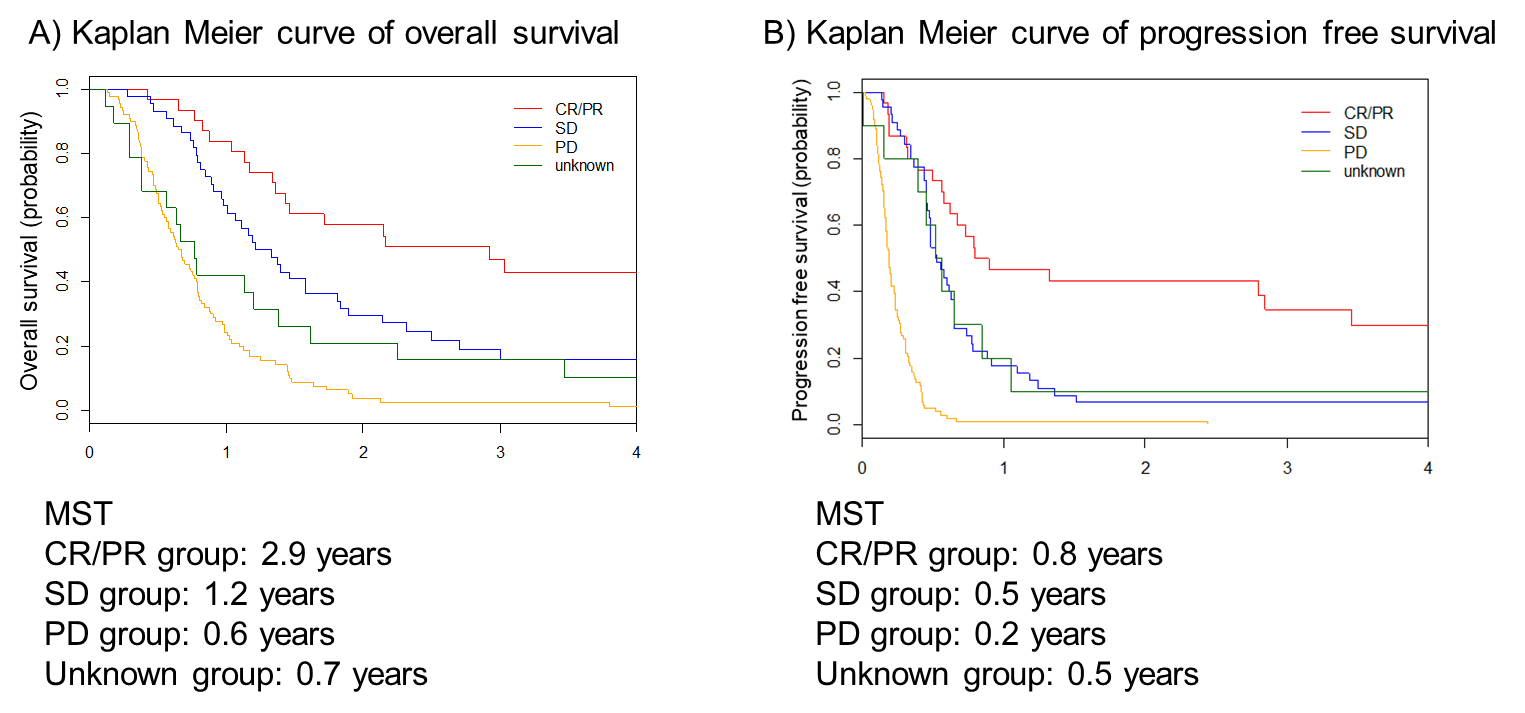


Table A1. Summary of treatments received

|  | | Original data set | Matched data set |
| --- | --- | --- | --- |
| **HT cohort** | | N = 99 | N = 71 |
| Median EDQ2 of definitive RT/CCRT, Gy | | 64.0 (35.1–81.0) | 68.1 (49.5–81.0) |
| ^a^ Time of diagnosis of persistent tumor, days | | 58 (0–276) | 51 (0–276) |
| ^b^ Treatment free interval, days | | 107 (13–393) | 98 (13–343) |
|  | |  |  |
| Treatments received | |  |  |
|  | HT alone | 62 (62.6) | 51 (72.9) |
|  | HT → ^c^ postoperative CT | 37 (37.4) | 20 (28.1) |
|  | |  |  |
|  | method of hysterectomy |  |  |
|  | simple hysterectomy | 60 (60.6) | 37 (52.1) |
|  | Piver-Rutledge type II radical hysterectomy | 9 (9.1) | 7 (9.9) |
|  | Piver-Rutledge type III radical hysterectomy | 24 (24.2) | 23 (32.4) |
|  | unknown | 6 | 4 (5.6) |
|  | |  |  |
|  | resection of lymph node |  |  |
|  | yes | 40 (40.4) | 34 (47.9) |
|  | no | 59 (59.6) | 37 (52.1) |
|  | |  |  |
| **CT cohort** | | N = 199 | N = 71 |
| Median EQD2 of definitive RT/CCRT, Gy | | 62.9 (20.8–86.0) | 64.0 (35.4–74.0) |
| ^a^ Time of diagnosis of persistent tumor, days | | 70 (0–348) | 50 (0–230) |
| ^b^ Treatment free interval, days | | 121 (11–425) | 96 (13–265) |
|  | |  |  |
| Treatments received | |  |  |
|  | CT regimen |  |  |
|  | taxane + platinum | 141 (71.0) | 50 (70.5) |
|  | irinotecan+platinum | 14 (7.0) | 5 (7.0) |
|  | 5FU+CDDP | 3 (1.5) | 0 |
|  | the other platinum combination | 16 (8.0) | 7 (9.9) |
|  | irinotecan+mitomycin C | 2 (1.0) | 0 |
|  | CDDP alone | 7 (3.5) | 3 (4.2) |
|  | nedaplatin alone | 2 (1.0) | 1 (1.4) |
|  | paclitaxel alone | 5 (2.5) | 1 (1.4) |
|  | irinotecan alone | 3 (1.5) | 1 (1.4) |
|  | unknown | 6 (3.0) | 3 (4.2) |
|  | |  |  |
|  | HT after systemic CT |  |  |
|  | ^d^ yes | ^e^ 6 (3.0) | ^e^ 14 (19.7) |
|  | no | 191 (96.0) | 55 (77.5) |
|  | unknown | 2 (1.0) | 2 (2.8) |

Number (%), or median (range) is shown.

^a^ Period from the date of completion of definitive RT to the date of diagnosis of persistent cervical cancer.

^b^ Period from the date of completion of definitive RT to the date of salvage hysterectomy or systemic chemotherapy.

Abbreviations: HT, hysterectomy; CT, chemotherapy; RT, radiotherapy; CCRT, concurrent chemoradiotherapy; HT, hysterectomy; 5FU, fluorouracil, CDDP, cisplatin and MMC, mitomycin C.

^c^ The decision to offer these patients systemic chemotherapy was made at each institution’s discretion.

^d^ The decision to offer these patients surgery was made after systemic chemotherapy.

^e^ The number in the original data set is less than in the matched data set because subjects could be restored and extracted in the propensity score-matching system.

Table A2. Adverse events for systemic chemotherapy

|  | Adverse Events | |
| --- | --- | --- |
|  | Any grade | Grade 3/4 |
| neutropenia (n=65) | 43 (66.2) | 26 (40) |
| anemia (n=65) | 50 (76.9) | 13 (20) |
| thrombocytopenia (n=65) | 22 (33.8) | 4 (6.2) |
| febrile neutropenia (n=65) | 2 (3.1) | 1 (1.5) |
| anorexia (n=54) | 42 (77.8) | 1 (1.9) |
| nausea (n=54) | 49 (90.7) | 1 (1.9) |
| vomiting (n=65) | 5 (9.8) | 0 (0) |
| diarrhea (n=59) | 12 (20.3) | 0 (0) |
| constipation (n=62) | 6 (9.7) | 0 (0) |
| alopecia (n=41) | 24 (58.5) | 0 (0) |
| peripheral sensory neuropathy (n=58) | 35 (60.3) | 3 (5.2) |
| peripheral motor neuropathy (n=45) | 0 (0) | 0 (0) |
| mucositis oral (n=47) | 2 (4.3) | 0 (0) |
| creatinine increased (n=64) | 17 (26.6) | 4 (6.3) |
| AST/ALT increased (n=64) | 5 (7.8) | 0 (0) |

Number (%) is shown.

Table A3. Evaluation of CT, MRI and PET-CT for persistent tumors after definitive RT/CCRT

| CT | Histologic findings after hysterectomy | |  |
| --- | --- | --- | --- |
| (n=33) | persistent tumor | no persistent tumor |  |
| Persistent tumor suspected (n=18) | 17 | 1 | PPV= 94.4% |
| No persistent tumor suspected (n=15) | 11 | 4 | NPV= 26.7% |
|  | Sensitivity =60.7% | Specificity=80.0% |  |
|  | | | |
| MRI | Histologic findings after hysterectomy | |  |
| (n=60) | persistent tumor | no persistent tumor |  |
| Persistent tumor suspected (n=54) | 46 | 8 | PPV= 85.2% |
| No persistent tumor suspected (n=6) | 4 | 2 | NPV= 33.3% |
|  | Sensitivity =92.0% | Specificity=20.0% |  |
|  | | | |
| PET-CT | Histologic findings after hysterectomy | |  |
| (n=45) | persistent tumor | no persistent tumor |  |
| Persistent tumor suspected (n=40) | 33 | 7 | PPV= 82.5% |
| No persistent tumor suspected (n=5) | 5 | 0 | NPV= 0% |
|  | Sensitivity =86.8% | Specificity=0% |  |

Table A4. Sites of progressive disease after salvage HT or systemic CT.

|  | salvage HT group (n=71) | systemic CT group (n=71) | *p* value |
| --- | --- | --- | --- |
| Progression | 38 (53.5%) | 55 (77.4%) | 0.003 |
| Sites of progressive disease |  |  |  |
| Local | 14 (19.7%) | 24 (33.8%) |  |
| Distance | 17 (23.9%) | 10 (14.1) |  |
| Both | 7 (9.9%) | 17 (23.9%) |  |
| unknown | 0 | 4 (5.6%) |  |

Table A5. Participating institutions and sample size.

| **Site** | **Number** |
| --- | --- |
| Osaka University Graduate School of Medicine., Osaka | 34^#^ |
| Graduate School of Medical Science, University of the Ryukyus, Okinawa | 22 |
| Saitama Cancer center, Saitama | 22 |
| Kanagawa Cancer Center, Kanagawa | 21 |
| Shizuoka Cancer Center, Shizuoka | 21 |
| Faculty of Medicine, University of Tsukuba, Ibaraki | 19 |
| Graduate School of Medicine, Chiba University, Chiba | 16 |
| Hyogo Cancer Center, Hyogo | 15 |
| Nagoya University Graduate School of Medicine, Aichi | 12 |
| Aichi Cancer Center Hospital, Aichi | 11 |
| Kurume University School of Medicine, Kurume | 10 |
| Tohoku University Hospital, Miyagi | 10 |
| Osaka International Cancer Institute, Osaka | 8 |
| The Jikei University Hospital, Tokyo | 8 |
| Hokkaido University Hospital, Hokkaido | 7 |
| National Cancer Center Hospital, Tokyo | 7 |
| Tokyo Metropolitan Cancer and Infectious Disease Center Komagome Hospital, Tokyo | 7 |
| Cancer Institute Hospital, Tokyo | 7 |
| Osaka City University Hospital, Osaka | 7 |
| Kyoto University Hospital, Kyoto | 6 |
| Keio University Hospital | 5 |
| Niigata University Medical and Dental Hospital, Niigata | 5 |
| Ehime University Hospital, Ehime | 5 |
| Iwate Medical University Hospital, Iwate | 4 |
| Juntendo University Hospital, Tokyo | 4 |
| Niigata Cancer Center Hospital, Niigata | 4 |
| NHO Shikoku Cancer Center, Ehime | 4 |
| The Jikei University Kashiwa Hospital, Chiba | 3 |
| NHO Kyushu Cancer Center, Fukuoka | 3 |
| Sapporo Medical University Hospital, Hokkaido | 2 |
| National Defense Medical College Hospital, Saitama | 2 |
| Osaka Medical College Hospital, Osaka | 2 |
| NHO Kure medical Center and Chugoku Cancer Center, Hiroshima | 2 |
| Gunma Prefectural Cancer Center, Gunma | 1 |
| Kagoshima University Hospital, Kagoshima | 1 |
| Total | 317 |

Number of cases is shown per participating institution listed in descending order.

#: some of these subjects were included in previous studies [1-2] regarding treatment after radiotherapy or concurrent chemoradiotherapy in Osaka University Hospital.

1. Mabuchi S, Kozasa K, Kimura T: Radical hysterectomy after radiotherapy for recurrent or persistent cervical cancer. Int J Gynaecol Obstet 139: 185-191, 2017
2. Mabuchi S, Matsumoto Y, Komura N, et al: The efficacy of surgical treatment of recurrent or persistent cervical cancer that develops in a previously irradiated field: a monoinstitutional experience. Int J Clin Oncol 22: 927-936, 2017
